# Peaks in online inquiries into pharyngitis-related symptoms correspond with annual incidence rates

**DOI:** 10.1007/s00405-020-06362-4

**Published:** 2020-09-23

**Authors:** Faris F. Brkic, Gerold Besser, Stefan Janik, Anselm J. Gadenstaetter, Thomas Parzefall, Dominik Riss, David T. Liu

**Affiliations:** Department of Otorhinolaryngology, Head and Neck Surgery, Medical University of Vienna, Währinger Gürtel 18-20, 1090 Vienna, Austria

**Keywords:** Google trends, Infodemiology, Acute pharyngitis, Cosinor, Pharyngitis, Sore throat

## Abstract

**Objective:**

To assess whether web-based public inquiries into pharyngitis-related search terms follow annual incidence peaks of acute pharyngitis in various countries from both hemispheres.

**Methods:**

Google Trends (GT) was utilized for systematic acquisition of pharyngitis-related search terms (sore throat, cough, fever, cold). Six countries from both hemispheres including four English (United Kingdom, United States, Canada, and Australia) and two non-English speaking countries (Austria and Germany) were selected for further analysis. Time series data on relative search interest for pharyngitis-related search terms, covering a timeframe between 2004 and 2019 were extracted. Following reliability analysis using the intra-class correlation coefficient, the cosinor time series analysis was utilized to determine annual peaks in public-inquiries.

**Results:**

The extracted datasets of GT proved to be highly reliable with correlation coefficients ranging from 0.83 to 1.0. Graphical visualization showed annual seasonal peaks for pharyngitis-related search terms in all included countries. The cosinor time series analysis revealed these peaks to be statistically significant during winter months (all *p* < 0.001).

**Conclusion:**

Our study revealed seasonal variations for pharyngitis-related terms which corresponded to winter incidence peaks of acute pharyngitis. These results highlight the need for easily accessible information on diagnosis, therapy, and red-flag symptoms for this common disease. Accurately informed patients might contribute to a reduction of unnecessary clinic visits and potentially cutback the futile antibiotic overuse.

**Electronic supplementary material:**

The online version of this article (10.1007/s00405-020-06362-4) contains supplementary material, which is available to authorized users.

## Introduction

Acute pharyngitis is one of the most prevailing conditions in otorhinolaryngology with an annual prevalence of about 40% in pediatric patients and 15% in adults. The most commonly reported pharyngitis-related symptoms are sore throat [1], followed by fever, headache, enlarged cervical lymph nodes, and cough [2]. Acute pharyngitis is mostly caused by viral infections and often requires only symptomatic therapy [1]. Yet, only 10–15% of cases are related to bacterial infections which warrant further antibiotic therapy [3]. Nevertheless, overtreatment with antibiotics is common and frequently caused by misinformed patients hoping for rapid pain relief and fast recovery. This overtreatment and other reasons, such as availability of antibiotics without prescription, contribute to the global issue of antibiotic resistance [4]. Moreover, acute pharyngitis also leads to a significant socioeconomic burden due to unnecessary ambulatory visits by misguided and misinformed patients [5].

The incidence of acute pharyngitis shows seasonal variations, with peaks during winter months. It may be caused by desiccated pharyngeal mucosa due to dry and cold air, making it friable and vulnerable to infections with the higher number of respiratory viruses during the cold season [3]. Considering that the vast majority of teenagers and adults use the World Wide Web to acquire health-related information, we hypothesized that peaks in web-based internet searches for pharyngitis-related symptoms might also follow the global incidence rates of this condition. However, up to now, there is no literature available addressing this matter.

The internet has become the most important information source for people generally, and particularly in cases of health- and disease-related issues [6]. The most popular online search engine is Google, with about 70% of daily World Wide Web searches performed using this platform [7]. Google trends (GT) is a publicly available analysis tool that allows a keyword-driven analysis of a portion of Google searches performed [8]. Infodemiology is a newly proposed research area. It involves the analysis of online search patterns to gain more insight on human behavior to inform medical professionals [9]. Indeed, several medical reports assessed the seasonality of different symptoms and diseases, such as tinnitus, epistaxis, laryngitis or dengue fever using GT [9–15]. However, up to now, literature has been sparse on web-based inquiries regarding acute pharyngitis or its most common symptoms.

Therefore, the aim of this study was to assess web-based public interest for acute pharyngitis and related-terms for seasonal variations globally. Furthermore, the validity and reproducibility of data acquired using GT was analyzed. The results are discussed in terms of the necessity for clear, easily accessible, and accurate general information on acute pharyngitis.

## Materials and methods

### Google trends

GT (Google LLC) is an online, publicly accessible, search term analysis engine. It allows analysis of search-query volume (frequency) for search terms that were entered on Google web search. These searches can be grouped for geographical location, timeframe (dating back to 2004), category, and subject [9, 10]. The search frequency is displayed as the relative search volume (RSV), indicating the interest for a specified search-term. Furthermore, GT allows comparing the RSV of up to five different search-terms. Moreover, the “Related Queries” option of GT allows the exploration of search terms that users entered after searching for the targeted keyword [9].

### GT search approach

To assess and illustratively depict seasonal variations of global RSV for pharyngitis-related search terms, we included countries from both hemispheres. In line with previous studies, we selected Australia, Canada, the United Kingdom (UK), and the United States of America (USA) as English-speaking countries from both hemispheres [9, 10]. We selected Germany and Austria as non-English speaking countries from the northern hemisphere. To explore pharyngitis related search terms that Google users entered on Google web search to gain more insight into acute pharyngitis, we entered five different search terms related to pharyngitis and its country-specific translations on April 22nd 2020. For English speaking countries: [sore throat], [strep], [fever], [cold], [cough] and for non-English-speaking countries: [Erkältung], [Fieber], [Halsschmerzen], [Husten], [Schnupfen]. We then applied GT-option “Related queries” for each of the above-mentioned keywords and noted all related inquiries (Supplementary Table 1). In addition, we compared all related inquiries with one another to depict the five most relevant search terms. Since previous studies provided evidence that GT-data from countries with a lower number of inhabitants are less reliable [9, 10], we entered the five above-mentioned search terms on seven consecutive days for each country included, starting from April 22nd 2020. GT searches were specified for the following parameters: timeframe between January 1st, 2004 until December 31st, 2019 and “health” category. For interpretation of seasonal patterns, winter months of the northern hemisphere were defined as January, February, and March, while summer months were defined as June, July, August, and September. Winter and summer months were defined vice versa for the southern hemisphere.

### Statistical analysis

Data were analyzed using the “season” and “psych” package in R 3.5.1 (R Development Core Team, 2008; R Foundation for Statistical Computing, Vienna, Austria). Following graphical visualization based on histograms, single-time series data were analyzed for reliability using the intraclass correlation coefficient (ICC_2,1_) [16]. Subsequently, annual seasonal variations in RSV were assessed for data retrieved from the first day of data extraction (April 22nd 2020) of time series data using the cosinor analysis. The exact model is described in more detail elsewhere [17]. Summed up, the cosinor model fits a sine wave to a predefined timeframe based on linear regression. Since we assessed annual variations, one peak was defined for every 12 months. The sinusoid is characterized based on a Phase (*P,* peak) and an Amplitude (*A*, size of the peak). Statistical significance of the model was tested with alpha level set at 0.025 to control for Type I errors.

## Results

### Pharyngitis-related search terms in countries from both hemispheres

First, we determined the most relevant pharyngitis-related search terms in English and non-English speaking countries from both hemispheres. We, therefore, applied GT-function “Related queries” and compared all pharyngitis-related inquiries with one another to explore the most relevant pharyngitis related search terms. The analyses revealed different relevant search terms in English and non-English speaking countries, which were used for subsequent analysis.

### Reliability analysis

We have previously shown that data from countries with less inhabitants show lower reliability compared to higher populated countries [9, 10]. We aimed to determine the reliability of GT-data which were assessed on seven consecutive days as mentioned above using the intraclass correlation coefficient (Tables  1,  2) (Supplementary Tables 2–5).

**Table 1:** Reliability of single and averaged time series data on common cold- related search terms in Australia

Search term	Measure	Intraclass correlation	Lower bound	Upper bound	*F*	Df1	Df2	*p* value
Cold	Single	0.98	0.97	0.98	387.5	191	1337	< .001
	Average	0.98	1.00	1.00	387.5	191	1337	< .001
Cough	Single	0.98	0.97	0.98	495.8	191	1337	< .001
	Average	1.00	1.00	1.00	495.8	191	1337	< .001
Fever	Single	0.79	0.75	0.82	34.8	191	1337	< .001
	Average	0.97	0.96	0.97	34.8	191	1337	< .001
Sore throat	Single	0.84	0.72	0.90	115.1	191	1337	< .001
	Average	0.98	0.95	0.99	115.1	191	1337	< .001
Strep	Single	0.72	0.68	0.76	21.4	191	1337	< .001
	Average	0.95	0.94	0.96	21.4	191	1337	< .001

*Single* single time series data, *average* averaged time series data, intraclass correlation intraclass correlation coefficient, *lower and upper bound* 95% confidence interval of the intraclass correlation coefficient, *F* *F* test for significance of the correlation coefficient, *Df1* numerator degrees of freedom, *Df2* denominator degrees of freedom

**Table 2:** Reliability of single and averaged time series data on common cold- related search terms in Austria

Search term	Measure	Intraclass correlation	Lower bound	Upper bound	*F*	Df1	Df2	*p* value
Erkältung	Single	0.95	0.94	0.96	163.3	191	1337	< .001
	Average	0.99	0.99	0.99	163.3	191	1337	< .001
Husten	Single	0.97	0.96	0.97	271.3	191	1337	< .001
	Average	1.00	1.00	1.00	271.3	191	1337	< .001
Fieber	Single	0.75	0.61	0.83	63.9	191	1337	< .001
	Average	0.96	0.93	0.98	63.9	191	1337	< .001
Halsschmerzen	Single	0.84	0.81	0.86	43.8	191	1337	< .001
	Average	0.98	0.97	0.98	43.8	191	1337	< .001
Schnupfen	Single	0.88	0.86	0.90	63.0	191	1337	< .001
	Average	0.98	0.98	0.99	63.0	191	1337	< .001

*Single* single time series data, *average* averaged time series data, *intraclass correlation* intraclass correlation coefficient, *lower and upper bound* 95% confidence interval of the intraclass correlation coefficient, *F* *F* test for significance of the correlation coefficient, *Df1* numerator degrees of freedom, *Df2* denominator degrees of freedom

Analysis revealed good to excellent reliability for all search terms. Search terms from countries with lower number of inhabitants such as Australia or Austria showed a slightly lower reliability compared to larger countries such as the USA or Germany.

### Seasonal peaks in search volume for pharyngitis-related search terms

**Fig. 1:**
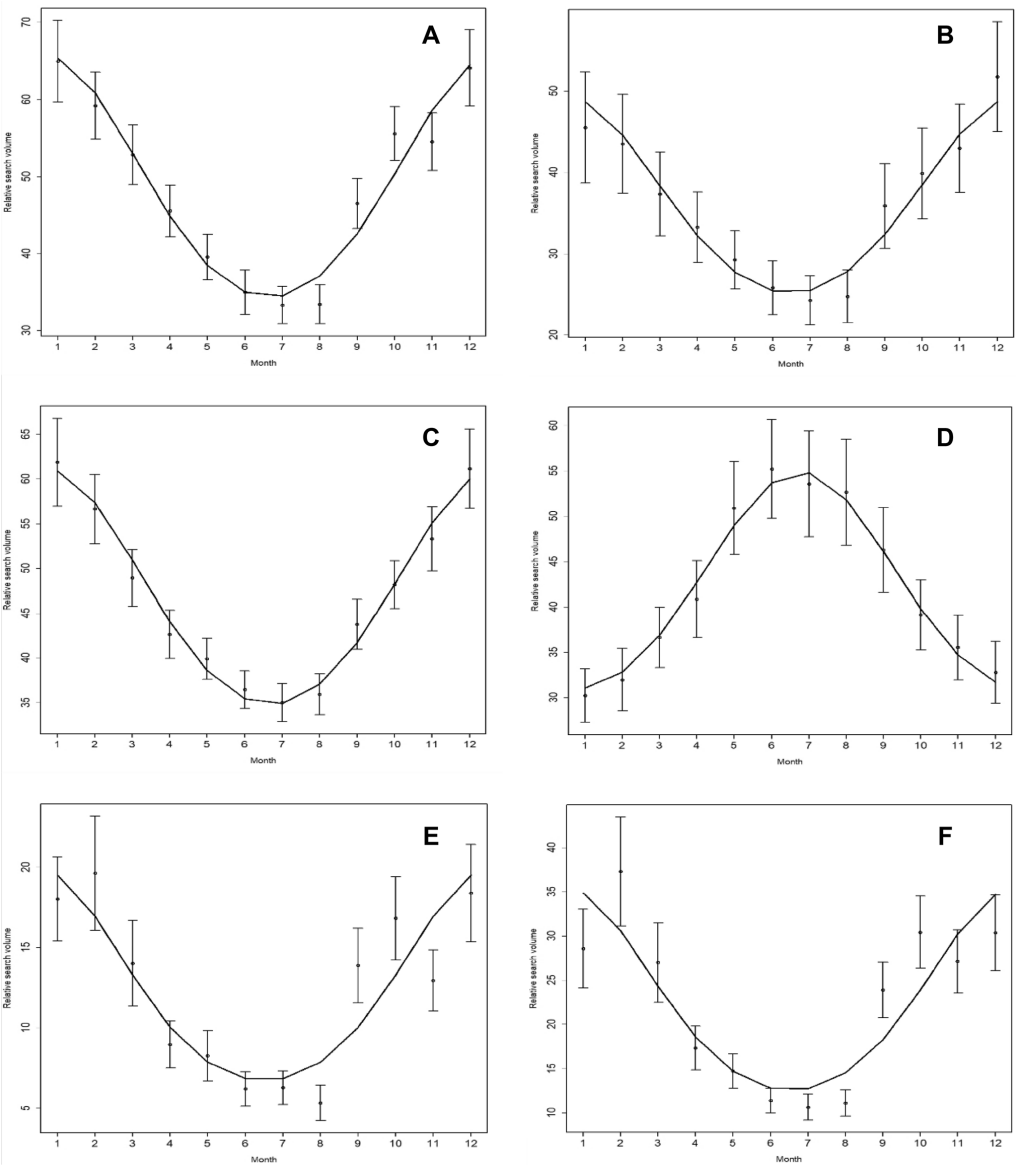
Cosinor model for seasonal variation in search volume for search terms [cold] in Canada (**a**), United Kingdom (**b**), United States of America (**c**) and Australia (**d**) and [Erkältung] in Austria (**e**) and Germany (**f**). The middle point marks the mean, the error bars represent the standard error.

We then sought to determine the peak month for public interest in sore throat-related search terms for single time series data queried from the first day of data-retrieval—April 22nd, 2020 (Tables  3,  4; Fig. 1).

**Table 3:** Cosinor analysis on seasonality of common cold related search terms in English speaking countries

Country	Term	Amplitude	Peak^a^	Nadir^a^	Standard error	*p* value
Australia	Cold	13.68	6.8	12.8	0.016	< .001
	Cough	13.68	6.8	12.8	0.016	< .001
	Fever	4.88	9.6	3.6	0.015	< .001
	Sore throat	2.61	8.7	2.7	0.029	< .001
	Strep	0.31	7.9	1.9	0.045	< .001
Canada	Cold	18.18	12.7	6.8	0.015	< .001
	Cough	9.46	12.9	6.9	0.020	< .001
	Fever	6.23	1.6	7.6	0.018	< .001
	Sore throat	1.54	1.6	7.6	0.028	< .001
	Strep	3.74	2.2	8.2	0.025	< .001
UK	Cold	14.02	12.5	6.5	0.017	< .001
	Cough	10.33	12.6	6.6	0.020	< .001
	Fever	11.09	3.9	9.9	0.018	< .001
	Sore throat	2.22	1.7	7.7	0.029	< .001
	Strep	1.09	2.6	8.6	0.041	< .001
USA	Cold	15.01	12.7	6.7	0.015	< .001
	Cough	10.7	12.8	6.8	0.019	< .001
	Fever	5.19	2.1	8.1	0.017	< .001
	Sore throat	2.1	1.6	7.6	0.028	< .001
	Strep	5.03	1.9	7.9	0.023	< .001

^a^Number corresponds to the respective month (i.e., 1 = January, 2 = February)

**Table 4:** Cosinor analysis on seasonality of common cold related search terms in German speaking countries

Country	Term	Amplitude	Peak^a^	Nadir^a^	Standard error	*p* value
Austria	Erkältung	8.32	12.5	6.5	0.030	< .001
	Husten	14.13	12.9	6.9	0.021	< .001
	Fieber	10.09	1.5	7.5	0.018	< .001
	Halsschmerzen	1.67	1.3	7.3	0.035	< .001
	Schnupfen	4.92	1.1	7.1	0.029	< .001
Germany	Erkältung	14.35	12.5	6.5	0.022	< .001
	Husten	11.53	1.2	7.2	0.022	< .001
	Fieber	8.35	1.7	7.7	0.019	< .001
	Halsschmerzen	2.33	1.5	7.5	0.032	< .001
	Schnupfen	3.62	12.8	6.8	0.034	< .001

^a^Number corresponds to the respective month (i.e., 1 = January, 2 = February)

Cosinor analysis revealed significant seasonal variations and peaks in winter months for all search terms of countries from both hemispheres. For English speaking countries of the northern hemisphere (number represents the corresponding month): *Canada* [Cold] = 12.8; [Cough] = 12.9; [Fever] = 1.6; [Sore throat] = 1.6; [Strep] = 2.2 (Fig. 1a); *United Kingdom* [Cold] = 12.5; [Cough] = 12.6; [Fever] = 3.9; [Sore throat] = 1.7; [Strep] = 2.6 (Fig. 1b); *United States of America* [Cold] = 12.7; [Cough] = 12.8; [Fever] = 2.1; [Sore throat] = 1.6; [Strep] = 1.9 (Fig. 1c). For English speaking countries of the southern hemisphere: *Australia* [Cold] = 6.8; [Cough] = 6.8; [Fever] = 9.6; [Sore throat] = 8.7; [Strep] = 7.9 (Fig. 1d). For non-English speaking countries of the northern hemisphere: *Austria* [Erkältung] = 12.5; [Husten] = 12.9; [Fieber] = 1.5; [Halsschmerzen] = 1.3; [Schnupfen] = 1.1 (Fig. 1e); *Germany* [Erkältung] = 12.5; [Husten] = 1.2; [Fieber] = 1.7; [Halsschmerzen] = 1.5; [Schnupfen] = 12.8 (Fig. 1f).

## Discussion

Acute pharyngitis is mostly caused by viral infections, which require only symptomatic therapy. However, some cases warrant antibiotic therapy due to a bacterial infection. The differentiation between these two conditions is fairly difficult for non-medical professionals, leading to a frequent over-treatment with antibiotics. This substantially contributes to the fact that acute pharyngitis is a significant burden on health care systems worldwide [5]. These issues highlight the need to assess web-based public inquiries into pharyngitis to improve the effectiveness of web-based information distribution to the general population. The current study revealed winter peaks in World Wide Web inquiries for pharyngitis-related terms in countries from both hemispheres. These peaks correspond to annual incidence cycles of acute pharyngitis [3]. Moreover, the analysis also revealed excellent reliability for GT-inquiries into pharyngitis-related search terms.

As mentioned above, an estimated 80% of acute pharyngitis cases are of viral etiology and require only symptomatic therapy. This symptomatic therapy includes systemic and local analgesic and anti-inflammatory medication. Yet, the use of antibiotics remains high, although often not necessary and inappropriate [4]. Regarding therapy options for acute pharyngitis, new treatment regimens were investigated recently. Essak et al. [18] analyzed the use of topical antibiotics, which resulted in no clear benefits. Another group investigated the use of local, low dose flurbiprofen for the management of acute sore throat [19]. They concluded that this treatment option represents a useful first-line therapy for symptomatic relief of patients with acute pharyngitis/sore throat. In addition, the authors proposed that this new therapy option may lead to a reduction of unnecessary antibiotic prescriptions. Treatment-related information can be easily obtained on the World Wide Web by medical professionals. However, patients that try to treat themselves might easily be overwhelmed by the wealth of information that is available online. Therefore, it is crucial to provide reliable, easily accessible, and publicly available online information on diagnosis, treatment, and red-flag symptoms of usually self-limiting medical conditions such as acute pharyngitis. For example, vital information on pharyngitis related red-flags requiring special attention and therapy would include worsening symptoms or (unilateral) neck swelling or trismus as a possible sign of tonsillitis with beginning complications [20]. As mentioned above, patients looking for rapid recovery and pain relief tend to self-manage their complaints inappropriately [4]. Due to high incidence rates, this could certainly contribute substantially to antibiotic overuse and resistance. Moreover, the phenomenon of prescription-free antibiotics was noted, which again supports patient’s self-care and self-(mis)management [21]. Purchasing prescription antibiotics from other countries is an already widely discussed topic [22]. Lower thresholds for prescribing antibiotics as well as issuing them without medical assessment promotes self-treatment even further [23]. Moreover, there are several online pharmacies registered in the UK that do not require prescriptions for antibiotics, some of them even allowing patient-driven decision for antibiotic dose, choice and quantity [24]. The so-called no-prescription websites certainly reflect a further negative side of the World Wide Web [25]. These aggravate the problem of acquiring prescription-free medication including antibiotics. Based on the above-mentioned issues, the importance of properly informed general public is further underlined, as well as the monitoring of these online services by law enforcement agencies and health initiatives.

Acute pharyngitis was the cause for about 13 million outpatient visits in a single year in the USA [26]. The economic burden of therapy and doctors’ visits are, therefore, enormous with annual costs of approximately 1.2 billion US dollars in the USA [5]. In contrast, annual healthcare costs in the USA for the much more prevalent and potentially more severe influenza type A and B infections ranged from 2 to 5.8 billion US dollars [27]. A possible cost reduction of 30% was estimated with reduced unnecessary visits to outpatient departments and further 7.3% when cutting the futile antibiotic therapy for patients with an acute pharyngitis [5]. Until now, no clear guidelines exist in regard to diagnostics and therapy of acute pharyngitis. However, European guidelines consider it a self-limiting disease that does not automatically require specific therapy except for symptomatic. Antibiotic therapy is recommended only in high-risk patients [28, 29].

The importance of patients being accurately informed about medical conditions has already been discussed extensively. Van der Velden [30] provided a new structured approach for the management of an acute sore throat. The authors noted that informed and educated patients are a significant step for empowering self-management. First, patient’s expectations and concerns as well as their opinion on antibiotic use should be identified. Second, the severity of the condition must be assessed. This involves identifying risk factors for adverse events, e.g., red flags, such as unilateral neck swelling or trismus for patients with sore throat/pharyngitis [20]. Indeed, one randomized controlled trial showed that well-tailored websites with information on basic medical conditions such as sore throat, fever, runny nose etc. led to a better understanding of these problems by the general public compared to standard online-available information [31]. Furthermore, they provided first evidence that this type of online information service can contribute to self-managing of minor medical symptoms.

Besides common pharyngitis symptoms such as sore throat, cough and fever [1, 2], we also included other search terms during the analysis. Common cold is an often-used umbrella term for acute infections of the upper respiratory tract including acute pharyngitis [32]. Similarly, incidence peaks for these medical conditions occur mostly during the winter months [33]. Based on this consideration, the search term “cold” was included in our analysis. Likewise, the search term “Erkältung” was included in the analysis of German-speaking countries. Furthermore, given the fact that viral pharyngitis is often misidentified and mistreated as a streptococcal tonsillitis [4], we included the term “strep” in our analysis.

In recent years, GT was often used for infodemiological assessment of different medical phenomena [9–15]. As shown by Kang et al. [34], GT has been proven to be a good complementary source for surveillance of annual influenza outbreaks and noted that GT could be used for detecting early signals of major outbreaks. A raised concern is, however, the lack of reliability assessment in these studies. Searches using GT delivered slightly different results when performed on different timepoints (e.g., days), which was already reported in another study [16]. We have previously shown that the number of inhabitants of assessed countries might be related to the reliability of GT searches. To overcome the bias in this regard, we performed GT searches on 7 consecutive days. We were able to show excellent reliability for GT-inquiries on pharyngitis-related search terms for all included countries. Moreover, our analysis provided further evidence that GT-data from larger countries were more reliable. This was in alignment to results of previous studies [9, 10]. Again, we point out the importance of reliability assessment of data retrieved using GT.

The recent COVID-19 pandemic led to a significant rise in online inquiries related to COVID-19 and related symptoms [35–37]. Public inquiries into coronavirus has already been assessed using GT [37]. It was demonstrated that the use of digital epidemiology (e.g., infodemiology) allows good surveillance of local COVID-19 outbreaks. To circumvent the bias regarding overlapping symptoms to those of an acute pharyngitis (sore throat, cough, fever etc. [38]), we did not perform the search beyond December 31st 2019. Telephone triage and phone consultations have gained increasing importance during the current COVID-19 pandemic [39]. In case of acute pharyngitis, patients could be informed on red flags and symptoms of acute bacterial tonsillitis or signs of a peritonsillar abscess, which would require further medical assessment. Furthermore, pharmacists may play an important role in providing patients with basic medical information and directing them to health care professionals when necessary [40]. This is crucial particularly for patients trying to self-manage certain medical conditions which tend to visit pharmacies looking for pain killers or antibiotics. Furthermore, pharmacists might also represent the only medical contact person for these patients, as many would not visit a doctor’s office.

Despite new findings of the current study, there are some limitations that warrant further discussion. First, the data was searched using only one online engine (GT), which could have resulted in a selection bias; this risk is, however, minimized by the fact that almost 70% of online searches are performed using Google [7]. Second, no demographic data such as gender or age of users as potential covariates are included in the data. It has been noted that younger individuals tend to perform online searches regarding medical conditions more frequently than older adults do [41]. This may have an influence on the probability of looking up symptoms, diagnosis or therapy based on online search engines. However, when compared to epidemiological studies, data gathered using infodemiological methods are more real time, extensive, and include area span and time. Furthermore, infodemiological methods also simplify the process of information retrieval and improve the efficiency of research.

## Conclusion

We revealed seasonal variations in World Wide Web-based inquiries for pharyngitis-related terms, which correspond to annual incidence rates of the acute pharyngitis. These findings underline the importance of accurate and easily accessible medical online information, in particular information regarding treatment options, diagnostic algorithms, and red-flag symptoms. This may represent a significant step towards the reduction of unnecessary clinic visits. Moreover, the antibiotic overuse could be scaled down, therefore, contributing to a reduction of individual and global antibiotic resistances.

## Electronic supplementary material

Below is the link to the electronic supplementary material.

Supplementary file1 (DOCX 43 kb)

## Data Availability

Not applicable.
