# Supplementary material for: Peaks in online inquiries into pharyngitis-related symptoms correspond with annual incidence rates
Source: Eur Arch Otorhinolaryngol. 2020 Sep 23;278(5):1653–60. doi: 10.1007/s00405-020-06362-4 (PMC7510767; doi:10.1007/s00405-020-06362-4)
Supplement: Supplementary file 1 — Supplementary file1 (DOCX 43 kb) [file 405_2020_6362_MOESM1_ESM.docx]

**SUPPLEMENTARY MATERIAL TO:**

Peaks in Online Inquiries into Pharyngitis-related symptoms correspond with annual Incidence Rates

Running title: Online Inquiries into Pharyngitis

Faris F. Brkic^1^, Gerold Besser^1^, Stefan Janik^1^, Anselm J. Gadenstaetter^1^, Thomas Parzefall^1^, Dominik Riss^1^, David T. Liu^1^

^1^ Department of Otorhinolaryngology, Head and Neck Surgery, Medical University of Vienna, Vienna, Austria

**Corresponding Author**

David T. Liu MD

Department of Otorhinolaryngology, Head and Neck Surgery

Medical University of Vienna

Währinger Gürtel 18-20, 1090 Vienna, Austria

Telephone: +43 1 40 400 33340

E-Mail: david.liu@meduniwien.ac.at

**Supplementary Table 1.** Country-specific search terms that were evaluated and compared with each other during this study.

**UNITES STATES OF AMERICA**

COLD

cold sore, cold sores, cold symptoms, cold stone, cold medicine, common cold, sinus cold, cold and flu, cold remedies, cold feet, cold hands, symptoms of cold, cold turkey, cold contagious, head cold, chest cold, cold stone creamery, medicine for cold, tylenol cold, cold sore herpes, cold virus, cold sore on lip, baby cold, best cold medicine

COUGH

whooping cough, cough syrup, dry cough, cough medicine, chest cough, cough drops, coughing, sore throat cough, cough remedies, medicine for cough, bronchitis cough, croup cough, croup, chronic cough, kennel cough, baby cough, toddler cough, cough remedy, pneumonia cough, kids cough, bad cough, phlegm cough, best cough medicine, persistent cough, cough suppressant

FEVER

fever symptoms, scarlet fever, high fever, temperature, fever temperature, yellow fever, fever rash, low fever, sore throat fever, cold fever, fever chills, symptoms of fever, fever adults, low grade fever, what is a fever, valley fever, dengue fever, rheumatic fever, rocky mountain fever, hay fever, fever blister, cat scratch fever, rocky mountain spotted fever, baby fever, fever in adults

SORE THROAT

Strep, strep throat, sore throat symptoms, sore throat cough, fever sore throat, throat pain, sore throat remedies, headache sore throat, sore swollen throat, sore throat medicine, sore throat and cough, throat infection, best for sore throat, symptoms of sore throat, sore throat flu, sore throat causes, strep symptoms, medicine for sore throat, strep throat symptoms, fever and sore throat, sore dry throat, allergies sore throat, sore throat tea, honey sore throat, remedies for sore throat

STREP

strep throat, strep symptoms, symptoms strep throat, symptoms of strep, sore throat, symptoms of strep throat, strep contagious, strep antibiotics, strep infection, strep throat contagious, strep throat signs, strep rash, strep throat fever, is strep contagious, strep test, group strep b, strep throat antibiotics, signs of strep, is strep throat contagious, strep throat infection, strep throat tonsils, throat infection, strep throat pictures, signs of strep throat, strep throat adults

**CANADA**

COLD

cold sore, cold sores, cold symptoms, cold flu, sinus cold, cold and flu, tylenol cold, common cold, cold remedies, cold feet, cold medicine, cold contagious, cold fx, advil cold, chest cold, symptoms of cold, head cold, cough and cold, cold hands, feeling cold, cold turkey, advil cold and sinus, cold virus, cold medication, cold flu symptoms

COUGH

dry cough, cough syrup, whooping cough, chest cough, cough medicine, cough remedies, coughing, phlegm cough, bad cough, croup cough, croup, cough remedy, bronchitis, toddler cough, bronchitis cough, mucus cough, kennel cough, kids cough, chronic cough, cough at night, baby cough, persistent cough, cough drops, sore throat and cough, asthma cough

FEVER

fever temperature, temperature, fever symptoms, scarlet fever, fever Celsius, fever Fahrenheit, high fever, yellow fever, what is a fever, baby fever, fever rash, celsius to Fahrenheit, fever children, children fever, fever in adults, dengue fever, fever in children, symptoms of fever, fever in Celsius, low fever, fever temp, toddler fever, hay fever, what is fever temperature, rheumatic fever

SORE THROAT

sore throat symptoms, strep throat, cough sore throat, sore throat fever, sore throat remedies, throat pain, headache sore throat, throat infection, sore throat remedy, cough and sore throat, dry sore throat, sore throat causes, strep throat symptoms, remedies for sore throat, swollen glands sore throat, sore throat and fever, swollen glands, sore throat medicine, sore throat and neck, sore throat one side, throat cancer, tea for sore throat, mono, mono sore throat, remedy for sore throat

STREP

strep symptoms, symptoms strep throat, strep contagious, sore throat, strep throat contagious, symptoms of strep, symptoms of strep throat, is strep contagious, strep infection, is strep throat contagious, strep throat antibiotics, throat infection, group b strep, mono, tonsillitis, strep test, signs of strep, strep throat signs, strep rash, signs of strep throat, throat pain, how contagious is strep, strep throat adults, group a strep, strep throat cough

**AUSTRALIA**

COLD

flu, cold flu, cold sore, cold and flu, cold sores, cold symptoms, hot cold, cold tablets, cold and flu tablets, head cold, cold feet, feeling cold, common cold, cold contagious, cold hands, codral, cold fever, cold remedies, cold virus, baby cold, symptoms of cold, codral cold and flu, cold turkey, cold medicine, cold flu symptoms

COUGH
whooping cough, dry cough, whooping cough vaccine, cough medicine, cough syrup, chesty cough, chest cough, coughing, whooping cough adults, croup, croup cough, baby cough, sore throat cough, persistent cough, persistent, whooping cough in adults, cough remedies, phlegm cough, asthma cough, cough remedy, cough at night, mucus cough, bronchitis cough, toddler cough, bad cough

FEVER

glandular fever, fever symptoms, hay fever, scarlet fever, dengue fever, symptoms glandular fever, yellow fever, ross river, ross river fever, fever temperature, temperature, symptoms of fever, fever rash, rheumatic fever, high fever, cold fever, hay fever symptoms, symptoms of glandular fever, fever in adults, baby fever, yellow fever vaccination, fever in children, typhoid fever, chills fever, what is a fever

SORE THROAT

sore throat symptoms, fever sore throat, sore throat headache, swollen throat, strep throat, throat infection, sore throat and cough, sore throat remedies, dry sore throat, swollen glands, sore throat swollen glands, sore throat remedy, sore throat cure, tonsillitis, sore throat gargle, sore tongue, sore throat causes, tonsils, sore throat runny nose, sore throat and headache, fever and sore throat, sore throat treatment, sore throat one side, throat cancer, sore tongue and throat

STREP

strep throat, sore throat, strep infection, strep throat symptoms, throat infection, tonsillitis, strep throat adults, strep throat infection, streptococcus, symptoms of strep throat, what is strep throat, group a strep, strep pyogenes, strep throat contagious, strep a infection, is strep throat contagious, strep b infection, strep throat rash, tonsillitis, strep pneumoniae, pharyngitis, antibiotics for strep throat, strep a symptoms, rheumatic fever

**UNITED KINGDOM**

COLD

cold sore, cold symptoms, cold sores, cold feet, feeling cold, cold and flu, cold hands, common cold, baby cold, head cold, symptoms of cold, nhs cold, chest cold, cold virus, cold remedies, cold and cough, cold turkey, cold sweats, cold flu symptoms, always cold, cold medicine, cold hands and feet, how long does a cold last, cold one, symptoms of a cold

COUGH
whooping cough, dry cough, chesty cough, chest cough, cough medicine, tickly cough, coughing, persistent cough, persistent, mucus cough, cough syrup, whooping cough vaccine, baby cough, phlegm cough, bad cough, cough at night, cough and cold, cough remedies, croup, cough nhs, kennel cough, croup cough, child cough, cough and sore throat, cough remedy

FEVER

scarlet fever, glandular fever, hay fever, fever symptoms, yellow fever, temperature, fever temperature, symptoms of fever, symptoms glandular fever, symptoms hay fever, hay fever symptoms, fever pain, high fever, scarlet fever rash, dengue fever, scarlet fever symptoms, fever in adults, hayfever, rheumatic fever, yellow fever vaccine, scarlett fever, fever in children, symptoms of glandular fever, curve fever, viral fever

SORE THROAT

cough sore throat, sore throat symptoms, sore swollen throat, sore throat headache, sore throat and cough, strep throat, throat infection, sore tongue, sore throat swollen glands, swollen glands, sore throat remedies, nhs sore throat, tonsillitis, sore dry throat, dry throat, best for sore throat, symptoms of sore throat, sore throat cure, sore throat causes, throat cancer, sore throat and tongue, cold and sore throat, sore ear and throat, sore mouth and throat, headache and sore throat

TONSILLITIS

is tonsillitis, symptoms tonsillitis, tonsillitis contagious, is tonsillitis contagious, tonsils, symptoms of tonsillitis, tonsillitis antibiotics, tonsillitis nhs, sore throat, bacterial tonsillitis, tonsil, viral tonsillitis, tonsillitis, tonsillitis pain, antibiotics for tonsillitis, strep throat, tonsillitis in adults, glandular fever, how long does tonsillitis last, tonsillitis treatment, penicillin, tonsillitis children, tonsillitis cure, tonsillitis cause, tonsillitis signs

**GERMANY**

ERKÄLTUNG

bei erkältung, gegen erkältung, erkältung hausmittel, erkältung baby, grippe erkältung, erkältung symptome, erkältung was tun, erkältung fieber, sauna erkältung, sport erkältung, schwangerschaft erkältung, medikamente erkältung, erkältung loswerden, erkältung kopfschmerzen, dauer erkältung, erkältung dauer, erkältung schnell loswerden, tee erkältung, was gegen erkältung, hausmittel gegen erkältung, was tun bei erkältung, inhalieren erkältung, globuli erkältung, erkältung ansteckend, ohrenschmerzen erkältung

FIEBER

wann fieber, fieber ab, fieber ab wann, fieber kind, fieber baby, fieber kleinkind, fieber symptome, fieber bei kindern, hohes fieber, fieber senken, temperatur fieber, fieber messen, temperatur, dengue fieber, husten fieber, kinder fieber, fieber kopfschmerzen, 40 fieber, fieber erwachsene, durchfall fieber, fieber durchfall, grippe fieber, erkältung fieber, wann hat man fieber, fieber ausschlag

HALSSCHMERZEN

bei halsschmerzen, gegen halsschmerzen, hausmittel halsschmerzen, husten halsschmerzen, erkältung halsschmerzen, halsschmerzen was tun, halsschmerzen schluckbeschwerden, schluckbeschwerden, starke halsschmerzen, fieber halsschmerzen, hals, kopfschmerzen halsschmerzen, Ohrenschmerzen halsschmerzen, hausmittel gegen halsschmerzen, tee halsschmerzen, husten und halsschmerzen, was tun gegen halsschmerzen, halsschmerzen schwangerschaft, mandelentzündung, gurgeln halsschmerzen, kind halsschmerzen, halsschmerzen loswerden, kinder halsschmerzen, halsschmerzen homöopathie, medikamente halsschmerzen

HUSTEN

gegen husten, hausmittel husten, baby husten, trockener husten, husten schmerzen, schleim husten, husten schnupfen, husten kind, kleinkind husten, schmerzen beim husten, bronchitis, husten auswurf, starker husten, husten nachts, husten halsschmerzen, blut husten, hausmittel gegen husten, reizhusten, husten kinder, husten und schnupfen, krupp, krupp husten, zwiebelsaft, husten bei kindern, husten zwiebelsaft

SCHNUPFEN

baby schnupfen, schnupfen husten, gegen schnupfen, erkältung schnupfen, erkältung, schnupfen hausmittel, schnupfen und husten, schnupfen was tun, schnupfen halsschmerzen, schnupfen symptome, schnupfen kleinkind, hausmittel gegen schnupfen, der schnupfen, globuli schnupfen, schnupfen globuli, was gegen schnupfen, homöopathie schnupfen, kopfschmerzen schnupfen, schnupfen bei baby, chronischer schnupfen, schnupfen loswerden, inhalieren, schnupfen inhalieren, schnupfen kinder, allergie schnupfen

**AUSTRIA**

ERKÄLTUNG

gegen erkältung, hausmittel erkältung, erkältung baby, grippe, hausmittel gegen erkältung, verkühlung, erkältung sauna, erkältung was tun, erkältung symptome, verlauf erkältung, hühnersuppe erkältung, hühnersuppe, was tun bei erkäœltung, erkältung ansteckend, was tun gegen erkältung, schüssler salze erkältung

FIEBER

wann fieber, fieber ab, fieber ab wann, baby fieber, kleinkind fieber, symptome fieber, hohes fieber, fieber senken, dengue fieber, fieber bei kindern, kinder fieber, temperatur fieber, hausmittel fieber, fieber messen, wann hat man fieber, fieber ausschlag, ab wann hat man fieber, fieber bei baby, fieber erwachsene, 3 tages fieber, fieber nach impfung, fieber bei kleinkindern, 3 tage fieber, gliederschmerzen

HALSSCHMERZEN

gegen halsschmerzen, hausmittel halsschmerzen, starke halsschmerzen, angina, halsweh, hausmittel gegen halsschmerzen, halsschmerzen schluckbeschwerden, halsschmerzen kind, lutschtabletten, lutschtabletten halsschmerzen, hausmittel bei halsschmerzen, halsschmerzen schnell loswerden, halsschmerzen schwangerschaft, kehlkopfentzündung, seitenstrangangina, was tun gegen halsschmerzen, halsschmerzen englisch, mandelentzündung, einseitige halsschmerzen

HUSTEN

husten hausmittel, gegen husten, baby husten, husten schleim, kleinkind husten, trockener husten, hausmittel gegen husten, bronchitis, husten kinder, starker husten, zwiebel husten, husten und schnupfen, reizhusten, schmerzen beim husten, husten bei kindern, hausmittel bei husten, bellender husten, was tun bei husten, hustensaft, husten mit schleim, husten und fieber, husten bei baby, husten schwangerschaft

SCHNUPFEN

gegen schnupfen, hausmittel schnupfen, schnupfen baby, husten, hausmittel gegen schnupfen, schnupfen inhalieren, globuli schnupfen, homöopathie schnupfen, schnupfen kleinkind, schnupfen bei baby, was tun gegen schnupfen, verstopfte nase, chronischer schnupfen, inhalieren bei schnupfen, schnupfen dauer, schnupfen schwangerschaft

**Supplementary Table 2.** Reliability of single and averaged time series data on common cold- related search terms in Canada. Abbreviations: Single = Single time series data, Average = Averaged time series data, Intraclass Correlation = Intraclass correlation coefficient, Lower and upper bound = 95% Confidence interval of the intraclass correlation coefficient, F = F-test for significance of the correlation coefficient, Df1 = numerator degrees of freedom, Df2= denominator degrees of freedom

| **Search term** | **Measure** | **Intraclass Correlation** | **Lower Bound** | **Upper Bound** | **F** | **Df1** | **Df2** | **p-value** |
| --- | --- | --- | --- | --- | --- | --- | --- | --- |
| Cold | Single | 0.98 | 0.98 | 0.99 | 569.9 | 191 | 1337 | <.001 |
|  | Average | 1.00 | 1.00 | 1.00 | 569.9 | 191 | 1337 | <.001 |
| Cough | Single | 0.98 | 0.98 | 0.98 | 445.8 | 191 | 1337 | <.001 |
|  | Average | 1.00 | 1.00 | 1.00 | 445.9 | 191 | 1337 | <.001 |
| Fever | Single | 0.96 | 0.95 | 0.96 | 190.9 | 191 | 1337 | <.001 |
|  | Average | 0.99 | 0.99 | 1.00 | 190.9 | 191 | 1337 | <.001 |
| Sore throat | Single | 0.83 | 0.71 | 0.89 | 11.7 | 191 | 1337 | <.001 |
|  | Average | 0.98 | 0.95 | 0.99 | 11.7 | 191 | 1337 | <.001 |
| Strep | Single | 0.88 | 0.86 | 0.90 | 61.8 | 191 | 1337 | <.001 |
|  | Average | 0.99 | 0.98 | 0.99 | 61.8 | 191 | 1337 | <.001 |

**Supplementary Table 3.** Reliability of single and averaged time series data on common cold- related search terms in Germany. Abbreviations: Single = Single time series data, Average = Averaged time series data, Intraclass Correlation = Intraclass correlation coefficient, Lower and upper bound = 95% Confidence interval of the intraclass correlation coefficient, F = F-test for significance of the correlation coefficient, Df1 = numerator degrees of freedom, Df2= denominator degrees of freedom

| **Search term** | **Measure** | **Intraclass Correlation** | **Lower Bound** | **Upper Bound** | **F** | **Df1** | **Df2** | **p-value** |
| --- | --- | --- | --- | --- | --- | --- | --- | --- |
| Erkältung | Single | 1.00 | 0.99 | 1.00 | 1993.9 | 191 | 1337 | <.001 |
|  | Average | 1.00 | 1.00 | 1.00 | 1993.9 | 191 | 1337 | <.001 |
| Husten | Single | 0.99 | 0.99 | 1.00 | 1621.6 | 191 | 1337 | <.001 |
|  | Average | 1.00 | 1.00 | 1.00 | 1621.6 | 191 | 1337 | <.001 |
| Fieber | Single | 0.99 | 0.99 | 1.00 | 1713.4 | 191 | 1337 | <.001 |
|  | Average | 1.00 | 1.00 | 1.00 | 1713.4 | 191 | 1337 | <.001 |
| Halsschmerzen | Single | 0.98 | 0.98 | 0.99 | 472.2 | 191 | 1337 | <.001 |
|  | Average | 1.00 | 1.00 | 1.00 | 472.2 | 191 | 1337 | <.001 |
| Schnupfen | Single | 0.98 | 0.97 | 0.98 | 336.3 | 191 | 1337 | <.001 |
|  | Average | 1.00 | 1.00 | 1.00 | 336.3 | 191 | 1337 | <.001 |

**Supplementary Table 4.** Reliability of single and averaged time series data on common cold- related search terms in the United Kingdom. Abbreviations: Single = Single time series data, Average = Averaged time series data, Intraclass Correlation = Intraclass correlation coefficient, Lower and upper bound = 95% Confidence interval of the intraclass correlation coefficient, F = F-test for significance of the correlation coefficient, Df1 = numerator degrees of freedom, Df2= denominator degrees of freedom

| **Search term** | **Measure** | **Intraclass Correlation** | **Lower Bound** | **Upper Bound** | **F** | **Df1** | **Df2** | **p-value** |
| --- | --- | --- | --- | --- | --- | --- | --- | --- |
| Cold | Single | 1.00 | 1.00 | 1.00 | 2606.9 | 191 | 1337 | <.001 |
|  | Average | 1.00 | 1.00 | 1.00 | 2606.9 | 191 | 1337 | <.001 |
| Cough | Single | 1.00 | 1.00 | 1.00 | 3023.7 | 191 | 1337 | <.001 |
|  | Average | 1.00 | 1.00 | 1.00 | 3023.7 | 191 | 1337 | <.001 |
| Fever | Single | 0.99 | 0.99 | 1.00 | 995.4 | 191 | 1337 | <.001 |
|  | Average | 1.00 | 1.00 | 1.00 | 995.4 | 191 | 1337 | <.001 |
| Sore throat | Single | 0.94 | 0.89 | 0.96 | 329.5 | 191 | 1337 | <.001 |
|  | Average | 0.99 | 0.99 | 1.00 | 329.5 | 191 | 1337 | <.001 |
| Strep | Single | 0.88 | 0.82 | 0.92 | 111.5 | 191 | 1337 | <.001 |
|  | Average | 0.98 | 0.97 | 0.99 | 111.5 | 191 | 1337 | <.001 |

**Supplementary Table 5.** Reliability of single and averaged time series data on common cold- related search terms in the United States of America. Abbreviations: Single = Single time series data, Average = Averaged time series data, Intraclass Correlation = Intraclass correlation coefficient, Lower and upper bound = 95% Confidence interval of the intraclass correlation coefficient, F = F-test for significance of the correlation coefficient, Df1 = numerator degrees of freedom, Df2= denominator degrees of freedom

| **Search term** | **Measure** | **Intraclass Correlation** | **Lower Bound** | **Upper Bound** | **F** | **Df1** | **Df2** | **p-value** |
| --- | --- | --- | --- | --- | --- | --- | --- | --- |
| Cold | Single | 0.99 | 0.99 | 1.00 | 1868.9 | 191 | 1337 | <.001 |
|  | Average | 0.94 | 0.93 | 0.97 | 1868.9 | 191 | 1337 | <.001 |
| Cough | Single | 1.00 | 1.00 | 1.00 | 2655.2 | 191 | 1337 | <.001 |
|  | Average | 1.00 | 1.00 | 1.00 | 2655.2 | 191 | 1337 | <.001 |
| Fever | Single | 0.99 | 0.98 | 0.99 | 773.1 | 191 | 1337 | <.001 |
|  | Average | 1.00 | 1.00 | 1.00 | 773.1 | 191 | 1337 | <.001 |
| Sore throat | Single | 0.95 | 0.90 | 0.97 | 616.5 | 191 | 1337 | <.001 |
|  | Average | 0.99 | 0.99 | 1.00 | 616.5 | 191 | 1337 | <.001 |
| Strep | Single | 0.98 | 0.97 | 0.98 | 437.5 | 191 | 1337 | <.001 |
|  | Average | 1.00 | 1.00 | 1.00 | 437.5 | 191 | 1337 | <.001 |
